# Changes in salivary analytes in cows due to the in vitro presence of feed

**DOI:** 10.1186/s12917-022-03371-9

**Published:** 2022-07-14

**Authors:** M. D. Contreras-Aguilar, P. J. Vallejo-Mateo, E. Lamy, J. J. Cerón, C. P. Rubio

**Affiliations:** 1 Interdisciplinary Laboratory of Clinical Analysis of the University of Murcia (Interlab-UMU), Department of Animal Medicine and Surgery, Veterinary School, Regional Campus of International Excellence Campus Mare Nostrum, University of Murcia, Campus de Espinardo, 30100 Espinardo, Murcia, Spain; 2 Department of Animal Medicine and Surgery, Veterinary School, Regional Campus of International Excellence Campus Mare Nostrum, University of Murcia, Campus de Espinardo, 30100 Espinardo, Murcia, Spain; 3 MED Mediterranean Institute for Agriculture, Environment and Development, IIFA Instituto de Investigação e Formação Avançada, University of Évora, Núcleo da Mitra, Apartado 94, 7006-554 Évora, Portugal; 4 Department of Animal and Food Science, School of Veterinary Science, Universitat Autònoma de Barcelona, 08193 Cerdanyola del Vallès, Barcelona, Spain

**Keywords:** Biomarkers, Cow, Feed contamination, Saliva, Sialochemistry

## Abstract

**Background:**

The effect in a sialochemistry profile of the presence of usually available feed in dairy cows was evaluated by an in vitro experiment. For this purpose, a pooled clean saliva from five healthy dairy cows was incubated five times with a standard feed based on a total mixed ration (F), wheat hay (H), and grass (G). The salivary panel was integrated by biomarkers of stress (cortisol -sCor-, salivary alpha-amylase -sAA-, butyrylcholinesterase -BChE-, total esterase -TEA-, and lipase -Lip-), immunity (adenosine deaminase -ADA-), oxidative status (Trolox equivalent antioxidant capacity -TEAC-, the ferric reducing ability of saliva -FRAS-, the cupric reducing antioxidant capacity -CUPRAC-, uric acid, and advanced oxidation protein products -AOPP-), and enzymes, proteins, and minerals of general metabolism and markers of liver, muscle, and renal damage (aspartate aminotransferase -AST-, alanine aminotransferase -ALP-, γ-glutamyl transferase -gGT-, lactate dehydrogenase -LDH-, creatine kinase -CK-, creatinine, urea, triglycerides, glucose, lactate, total protein, phosphorus, and total calcium).

**Results:**

Most of the evaluated analytes showed a coefficient of variations (CV) higher than 15% and/or significant changes compared with the clean saliva when feed was present. Some analytes, such as the oxidative status biomarkers (CV > 80%), AST (CV > 60%), or glucose (CV > 100%), showed significant changes with all the feed types tested. Others showed significant differences only with certain types of feed, such as LDH with F (CV > 60%) or triglycerides with F (CV > 100%) and H (CV > 95%). However, sCor or gGT remained unchanged (CV < 15%, *P* > 0.05) in all the treatments.

**Conclusions:**

The presence of feed can produce changes in most of the analytes measured in cows’ saliva, being of high importance to consider this factor when saliva is used as a sample to avoid errors in the interpretation of the results.

**Supplementary Information:**

The online version contains supplementary material available at 10.1186/s12917-022-03371-9.

## Background

Nowadays, saliva is a diagnostic fluid with a growing research interest to evaluate and monitor inadequate welfare or stress situations in veterinary science [1] since it can be obtained by non-invasive and non-painful techniques [2, 3] without specialized staff [4]. Particularly in livestock species such as dairy cows, the detection of poor animal welfare conditions is necessary since they produce decreases the productivity and meat quality, reducing sustainable meat production, the milk yield and quality, and increasing the prevalence of metabolic diseases and the release and virulence of infectious diseases due to an immune function drop [1, 5–7]. Therefore, the use of salivary biomarkers that evaluate these states could be helpfully in avoiding such undesirable situations. Indeed, recent studies in dairy cows have detected changes in salivary analytes in physiological conditions such as the peripartum period and in diseases such as mastitis and lameness [8–11].

However, changes in saliva color, which usually is due to the presence ﻿of feed in the oral cavity, can interfere with analytical determinations, mainly if spectrophotometric/colorimetric methods are employed. In addition, the feed composition could also potentially interfere with selected analytes. For example, salivary alpha-amylase (sAA) activity can increase in the presence of food with a high amount of carbohydrates in humans [12]. Recently, a study in horses showed that the presence of feed could modify the results obtained from a panel of analytes measured in saliva [13]. Despite this, no scientific evidence about the feed effect on salivary analytes measured in the cow has been reported to the author's best knowledge.

The possible interference due to the presence of feed in the analytes measured in saliva from livestock is an important question in order to make appropriate saliva analysis and interpretation. Thus, this study aimed to investigate the potential effect of the presence of different types of feed in dairy cows in selected salivary biomarkers previously validated in cows' saliva [10] by an in vitro experiment. This sialochemistry profile was integrated by biomarkers of stress (salivary cortisol -sCor-, sAA, butyrylcholinesterase -BChE-, total esterase -TEA-, and lipase -Lip-); immunity (adenosine deaminase -ADA-); oxidative status (Trolox equivalent antioxidant capacity -TEAC-, the ferric reducing ability of saliva -FRAS-, the cupric reducing antioxidant capacity -CUPRAC-, uric acid, and advanced oxidation protein products -AOPP-); and enzymes, proteins, and minerals of general metabolism and biomarkers of liver, muscle, and renal damage (aspartate aminotransferase -AST-, alanine aminotransferase -ALP-, γ-glutamyl transferase -gGT-, lactate dehydrogenase -LDH-, creatine kinase -CK-, creatinine, urea, triglycerides, glucose, lactate, total protein, phosphorus, and total calcium).

## Results

### Effects of different treatments in saliva

**Fig. 1:**
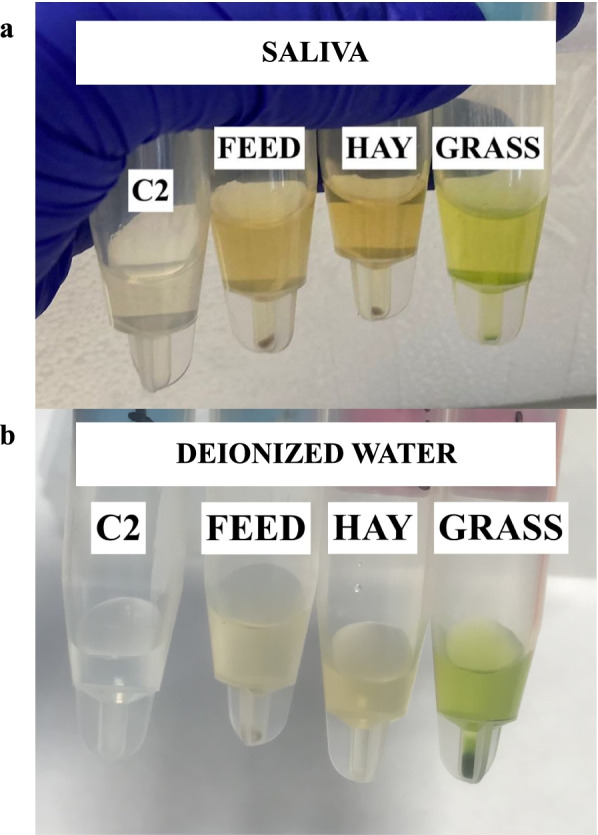
Color obtained after the centrifugation of the Salivette tubes once a cows’ saliva pool and deionized water were submitted to incubation during 5 min at 38 °C (C2) and with 250 mg of feed-based on a standard total mixed ration usually offered to lactation cows in production (**F**), wheat hay (**H**), and grass (**G**)

The different treatments (addition of a standard feed based on a total mixed ration = specimen F, wheat hay = specimen H, and grass = specimen G) in the in vitro experiment modified the original color from the clean saliva control (C1) (Fig. 1a).

**Fig. 2:**
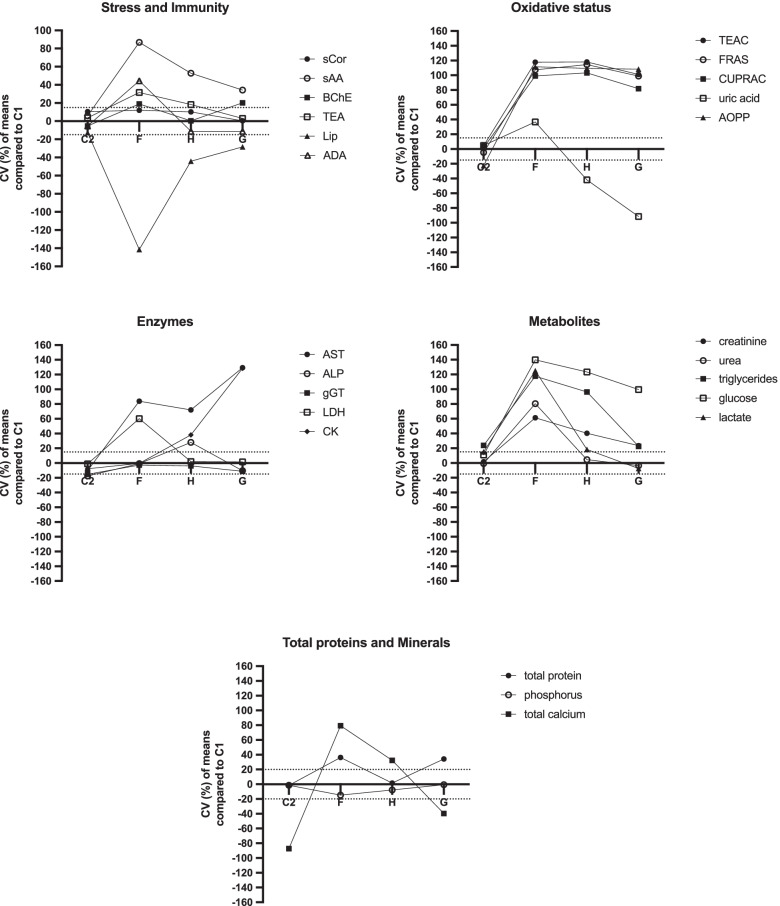
Coefficient of variations (CVs) of the means (*n* = 5) from the specimens C2 (cows’ saliva pool submitted to incubation during 5 min at 38 °C), F, H, and G (cows’ saliva pool submitted to incubation with 250 mg of feed-based a standard total mixed ration, wheat hay, and grass, respectively) compared to their control clean saliva pool (specimen C1)

Most of the analytes showed significant differences between C1 and after the different treatments (Table 1), and with variations higher than 15% of the intraassay coefficient of variation (CV) compared to the C1 results (Fig. 2). Those mainly were TEAC (mean difference with F = 0.527 mmol/L, CV = 117%; mean difference with H = 0.538 mmol/L, CV = 118%; and mean difference with G = 0.267 mmol/L, CV = 101%; *P* < 0.001), FRAS (mean difference with F = 0.638 mmol/L, CV = 107%; mean difference with H = 0.858 mmol/L, CV = 114%; and mean difference with G = 0.471 mmol/L, CV = 99%; *P* < 0.001), CUPRAC (mean difference with F = 0.462 mmol/L, CV = 99%; mean difference with H = 0.536 mmol/L, CV = 103%; and mean difference with G = 0.272 mmol/L, CV = 81%; *P* < 0.001), AOPP (mean difference with F = 383.3 µmol/L, CV = 112%; mean difference with H = 350.6, µmol/L CV = 109%; and mean difference with G = 335.6 µmol/L, CV = 108%; *P* < 0.01), AST (mean difference with F = 10.3 IU/L, CV = 84%; mean difference with H = 7.4 IU/L, CV = 72%; and mean difference with G = 75.4 IU/L, CV = 129%; *P* < 0.05), and glucose (mean difference with F = 3.91 mmol/L, CV = 140%; mean difference with H = 0.31 mmol/L, CV = 123%; and mean difference with G = 0.10 mmol/L, CV = 99%; *P* < 0.001).

**Table 1:** Means and standard deviation (SD) from five replicates of the measurements of the cows’ saliva specimens labeled C1 (clean saliva), C2 (clean saliva ﻿after incubation during 5 min at 38 °C), F (saliva with a total mixed ration usefully offered to lactation dairy cows), H (saliva with wheat hay), and G (saliva with grass); and of the ﻿deionized water specimens labeled C2_w_ (water after incubation during 5 min at 38 °C), F_w_ (water with a total mixed ration usually offered to lactation dairy cows), H_w_ (water with wheat hay), and G_w_ (water with grass)

		SALIVA						DEIONIZED WATER			
		C1	C2	F	H	G	*P* value^1^	C2_w_	F_w_	H_w_	G_w_
sCor (﻿µg/dL)	mean	0.094	0.109	0.116	0.109	0.094	.461	0.055^a^_a_	0.050^a^_a_	0.105	0.050^a^_a_
	SD	0.009	0.014	0.034	0.029	0.006		0.008	0.000	0.031	0.000
sAA (IU/L)	mean	1.2	1.3	4.8^b^	2.5^a^	1.9^a^	**.001**	0.2^a^	2.1_a_	0.9	1.1_a_
	SD	0.5	0.6	1.1	0.8	0.4		0.3	0.6	0.2	0.5
BChE (nmol/mL/min)	mean	15.3	14.7	21.4^a^	14.2	22.8^a^	**.017**	0.9^b^_a_	8.8^a^_b_	2.9^a^_b_	6.4^a^_a_
	SD	2.3	2.5	2.6	1.5	4.7		0.3	0.2	1.0	2.5
TEA (IU/L)	mean	80.1	84.3	126.0^c^	103.8^a^	83.5	**.006**	0.0^d^_d_	13.3^c^_b_	14.8^d^_b_	9.6^c^_b_
	SD	0.83	3.80	7.99	15.72	3.05		0.00	2.67	0.55	1.08
Lip (IU/L)	mean	5.7	3.9^a^	0.0^d^	2.5	3.1^b^	**.024**	3.2^a^_b_	0.0^d^	4.0	4.5
	SD	0.3	0.1	0.0	2.4	0.3		0.1	0.0	5.0	0.7
ADA (IU/L)	mean	2.2	2.0	4.2^a^	1.9	1.9^b^	**.003**	0.1^ cc^	4.7^b^	0.5^a^_a_	0.4^b^_a_
	SD	0.0	0.1	1.2	0.7	0.2		0.1	0.4	0.5	0.3
TEAC	mean	0.053	0.056	0.580^c^	0.591^c^	0.320^a^	** < .001**	0.000^d^	0.405^b^	0.410^b^	0.688^a^
(mmol/L)	SD	0.001	0.002	0.078	0.127	0.165		0.000	0.025	0.047	0.238
FRAS (mmol/L)	mean	0.101	0.095	0.739^c^	0.959^a^	0.572^c^	** < .001**	0.000^ cc^	0.512^c^_a_	0.690^a^	1.466^b^
	SD	0.003	0.002	0.095	0.218	0.293		0.000	0.016	0.077	0.650
CUPRAC (mmol/L)	mean	0.099	0.101	0.561^d^	0.635^c^	0.371^a^	** < .001**	0.047^d^_c_	0.407^d^_a_	0.485^b^	0.847^a^_a_
	SD	0.001	0.002	0.067	0.134	0.181		0.001	0.004	0.052	0.306
uric acid (µmol/L)	mean	6.12	6.49	9.71^c^	3.78^b^	1.10^a^	**.001**	5.12	2.38^b^_b_	0.00^b^_a_	0.00^b^
	SD	0.41	0.37	0.54	1.14	2.08		0.66	0.48	0.00	0.00
AOPP (µmol/L)	mean	51.3	37.2^b^	434.6^c^	401.9^b^	386.9^a^	**.002**	5.0^c^_b_	215.4^b^_a_	229.2^b^	514.3^a^
	SD	2.9	3.6	67.8	142.9	203.8		1.4	11.5	33.9	230.1
AST (IU/L)	mean	3.5	3.1	13.8^a^	10.9^a^	78.9^a^	**.021**	0.2^b^_b_	8.9^a^	28.7^b^_b_	322.5^a^_a_
	SD	0.6	0.2	5.6	4.3	44.8		0.3	2.9	5.2	160.3
ALP (IU/L)	mean	22.6	17.6^b^	22.4	33.8	19.5^b^	.086	1.4^ cc^	5.9^c^_b_	16.8_a_	4.5^b^_b_
	SD	0.7	0.4	2.0	13.5	0.9		0.2	1.5	8.3	2.1
gGT (IU/L)	mean	23.6	19.0^b^	22.6	22.3	20.1^a^	.063	1.5^ cc^	3.0^ cc^	2.5^c^_b_	2.4^ cc^
	SD	0.7	0.5	0.7	1.6	0.9		0.1	0.2	0.6	0.2
LDH (IU/L)	mean	24.2	23.8	60.0^b^	24.9	24.7	**.002**	4.2^b^_b_	54.3^a^	5.6^a^_a_	3.7^b^_b_
	SD	1.8	1.9	10.4	4.9	1.7		1.9	9.8	2.8	2.6
CK (IU/L)	mean	2.1	1.9^b^	2.1	3.6^a^	43.0^c^	**.001**	0.0^c^_b_	0.4^a^_a_	2.3	55.6^d^
	SD	0.07	0.09	0.23	0.76	8.97		0.00	0.32	0.10	0.52
creatinine (µmol/L)	mean	50.93	52.17	128.92^d^	91.43^b^	71.18^a^	** < .001**	0.59^ cc^	77.51^b^_b_	20.63^a^_b_	25.05^c^_a_
	SD	0.48	1.08	6.63	14.07	11.70		1.02	3.11	10.06	1.02
urea (mmol/L)	mean	3.45	3.41^c^	12.53^d^	3.68^a^	3.28^b^	**.002**	0.00^b^_b_	11.92^c^	0.13^a^_c_	0.00^b^_b_
	SD	0.06	0.14	0.72	0.37	0.37		0.00	1.71	0.11	0.00
triglycerides (mmol/L)	mean	0.13	0.18^c^	1.41^d^	0.68^a^	0.18^b^	**.002**	0.06^b^_b_	1.38^c^	0.36	0.11_a_
	SD	0.00	0.01	0.12	0.40	0.01		0.01	0.04	0.15	0.02
glucose (mmol/L)	mean	0.02	0.03^a^	3.93^d^	0.33^a^	0.13	** < .001**	0.00^a^	4.18^a^	0.30^a^	0.50^a^_a_
	SD	0.00	0.00	0.25	0.19	0.10		0.00	0.97	0.14	0.15
lactate (mmol/L)	mean	0.28	0.35	4.57^d^	0.37	0.25	**.001**	0.03^b^_a_	4.71^d^	0.09^b^_a_	0.02^b^_a_
	SD	0.00	0.06	0.28	0.09	0.04		0.02	0.99	0.03	0.03
total protein (g/L)	mean	0.52	0.51	0.89^c^	0.54	0.86^a^	**.014**	0.01^ cc^	0.12^b^_b_	0.04^c^_b_	0.80
	SD	0.01	0.02	0.06	0.03	0.25		0.00	0.03	0.01	0.40
phosphorus (mmol/L)	mean	7.70	7.55	6.25^b^	6.90^c^	7.64	** < .001**	0.00^ cd^	2.38^b^_b_	0.26^c^_b_	2.24^b^_b_
	SD	0.13	0.08	0.34	0.31	0.13		0.00	0.20	0.13	0.51
total calcium (mmol/L)	mean	0.33	0.08^b^	1.18^d^	0.53	0.19^a^	** < .001**	0.00^a^_b_	3.40^b^_a_	0.79	0.93^a^_a_
	SD	0.08	0.01	0.07	0.17	0.06		0.00	0.51	0.37	0.23

sCor = Salivary cortisol; sAA = salivary alpha-amylase; BChE = butyrylcholinesterase; TEA = total esterase; Lip = lipase; ADA = adenosine deaminase; TEAC = Trolox equivalent antioxidant capacity; FRAS = The ferric reducing ability of saliva; CUPRAC = The cupric reducing antioxidant capacity; AOPP = Advanced oxidation protein products; AST = Aspartate aminotransferase; ALP = Alanine aminotransferase; gGT = γ-glutamyl transferase; LDH = Lactate dehydrogenase; CK = Creatine kinase

^1^One-way repeated measures analysis of variance (ANOVA) study to evaluate if there were differences in the values obtained between the saliva specimens C1 (clean control saliva), C2, F, H, and G

^a,b,c,d^ Upper letters show statistical results between the saliva specimens (C2, F, H, and G) with C1 in uncorrected Fisher’s least significant difference (LSD) test for multiple comparisons: a: *P* < .05; b: *P* < .01; c: *P* < .001; d: *P* < .0001

_a,b,c,d_ Down letters show statistical results between the water specimens (C2_w_, F_w_, H_w_, and G_w_) with their homologous with saliva in uncorrected Fisher’s least significant difference (LSD) test for multiple comparisons: a: *P* < .05; b: *P* < .01; c: *P* < .001; d: *P* < .0001

The analytes that did not show these variations were sCor (mean difference = 0.022 µg/dL, CV = 12%, *P* = 0.100), ALP (mean difference = –0.2 IU/L, CV = 1%, *P* = 0.757), gGT (mean difference = –1.0 IU/L, CV = 3%, *P* = 0.088), and CK (mean difference = 0.0 IU/L, CV = 0%, *P* > 0.999) in the treatment F; sCor (mean difference = 0.015 µg/dL, CV = 10%; *P* = 0.406), BChE (mean difference = –1.0 nmol/mL/min, CV = 1%, *P* > 0.999), ADA (mean difference = –0.3 IU/mL, CV = 11%, *P* = 0.392), gGT (mean difference = –1.3 IU/mL, CV = 4%, *P* = 0.181), LDH (mean difference = 0.7 IU/mL, CV = 2%, *P* = 0.389), and total protein (mean difference = 0.02 g/L, CV = 2%, *P* = 0.428) in the treatment H; and sCor (mean difference = 0.000 µg/dL, CV = 0%, *P* > 0.999), TEA (mean difference = 3.4 IU/L, CV = 3%, *P* = 0.085), gGT (mean difference = –3.5 IU/mL, CV = 4%, *P* = 0.05), LDH (mean difference = 0.5 IU/mL, CV = 2%, *P* = 0.248), lactate (mean difference = –0.03 mmol/L, CV = 7%, *P* = 0.235), and phosphorus (mean difference = –0.06 mmol/L, CV = 1%, *P* = 0.508) in the treatment G.

The clean saliva specimen incubation at 38ºC (C2) produced significant changes compared to C1 (Fig. 2) in the levels of AOPP (CV = –22.5%), ALP (CV = –17.6%), triglycerides (CV = 23.8%), and total calcium (CV = –87.3%).

### Effects of the different treatments in water

The presence of F, H and G added color on the water deionized after the different treatments in the in vitro experiment (Fig. 1b) with lower intensity than on saliva (Fig. 1a).

The values in the water specimens compared to their homologs in saliva (F vs. F_w_, H vs. H_w_, and G vs. G_w_,Table 1) were not statistically different for the treatment F in Lip (mean difference = 0.0 IU/L, *P* > 0.999), ADA (mean difference = 0.5 IU/L, *P* = 0.461), TEAC (mean difference = –0.175 mmol/L, *P* = 0.061), AST (mean difference = –4.9 IU/L, *P* = 0.152), LDH (mean difference = –5.7 IU/L, *P* = 0.583), urea (mean difference = –0.61 mmol/L, *P* = 0.681), triglycerides (mean difference = –0.03 mmol/L, *P* = 0.681), glucose (mean difference = 0.25 mmol/L, *P* = 0.709), and lactate (mean difference = 0.14 mmol/L, *P* = 0.860); for the treatment H in Lip (mean difference = 1.5 IU/L, *P* = 0.607), TEAC (mean difference = –0.181 mmol/L, *P* = 0.117), FRAS (mean difference = –0.269 mmol/L, *P* = 0.102), CUPRAC (mean difference = –0.150 mmol/L, *P* = 0.169), AOPP (mean difference = –172.7 µmol/L, *P* = 0.129), triglycerides (mean difference = –0.32 mmol/L, *P* = 0.223), glucose (mean difference = –0.03 mmol/L, *P* = 0.792), and total calcium (mean difference = 0.26 mmol/L, *P* = 0.154); and for the treatment G in Lip (mean difference = 1.4 IU/L, *P* = 0.607), TEAC (mean difference = 0.368 mmol/L, *P* = 0.117), FRAS (mean difference = 0.894 mmol/L, *P* = 0.102), and AOPP (mean difference = 127.4 µmol/L, *P* = 0.129).

## Discussion

This study evaluated the effect of feed on a sialochemistry profile in dairy cows in order to provide scientific evidence about the conditions in which the saliva should be obtained in this species. The analytes included in this profile are biomarkers of stress, poor welfare, or health status [9, 10]. In humans, it is recommended to perform the saliva sampling procedure after rinsing the individuals’ mouths to avoid any analytical measurement interferences due to the presence of food particles [14, 15]. In horses, the use of clean saliva is also advised to evaluate biomarkers in saliva [13]. Therefore, these authors hypothesized that the analytes evaluated in this sialochemistry profile could be altered due to the feed presence in cows. However, to the author’s best knowledge, there is no scientific evidence about this effect in measuring analytes in dairy cows’ saliva.

Our results confirmed that most analytes included in the sialochemistry profile in dairy cows were affected by the presence of the feed usually available for this species. These changes could be due to various reasons. One would be the feed composition. For example, the broccoli remains in the total mixed ration [16], the wheat hay, and the grass have a per se antioxidant capacity [17, 18], and their its content in glucose, fats, and minerals, among others, may cause several interferences in the analyte assays measured as previously observed in human [19]. A second reason could be the color/turbidity change when adding the feed, as observed in Fig. 1, which can also interfere with the analytical measurements, especially those measured by spectrophotometric/colorimetric methods [20]. In addition, changes in some analytes, such as AOPP, ALP, triglycerides, and total calcium, in the clean saliva control 2 (C2) could be due to the effect of the incubation at 38ºC. For example, the total calcium concentrations in that warmed clean saliva (C2) were much lower (mean concentration = 0.08 ± 0.01 mmol/L) than in the clean control saliva (C1, mean concentration = 0.33 ± 0.08 mmol/L). It has been postulated that the salivary components and enzymes (i.e., sAA) can use the initial calcium from saliva for their activities after being activated by the temperature [21], reducing the total available calcium concentration in the sample. However, further studies should evaluate the mechanisms that can produce these analytical variations in cow saliva.

The changes occurring in our study due to the presence of feed in cow saliva could affect the clinical interpretation of selected analytes when assessing pathological/stressful conditions, as is the case of the Lip, oxidative status biomarkers, AST, triglycerides, glucose, or lactate measured in saliva with all or some of the feeds evaluated in this study, since their values changed by around 100% compared to the clean saliva. However, there were other salivary analytes showing significant changes when the saliva is contaminated with feed, but whose changes would not be clinically relevant. For instance, ADA with the total mixed ration increased from 2.2 IU/L to 4.2 IU/L; however, it is reported median values of 22.4 IU/L ﻿within the 12 first hours after calving, a situation in which there is an inflammatory component, compared to median values of 6.1 IU/L before calving [22]. In addition, there were analytes that showed no significant changes and/or variations lower than 15% of the CV of the analyte when some of the feeds were added. This occurred in the sCor, ALP, gGT, phosphorus, ADA in the presence of wheat hay and/or grass, CK in the total mixed ration, or lactate in the grass. Therefore, these analytes would not be affected in cow saliva by the presence of the feed tested in our sutdy. This would be relevant since ADA, gGT, and lactate in saliva have been proposed as inflammatory biomarkers [8, 10, 23], and CK related to the milk yield in dairy cows.

In any case, it would be recommended as much as possible to obtain clean specimens when analytes are going to be measured in cows' saliva. When mouth washing is not possible, alternatives for obtaining clean saliva, such as sampling ﻿in the milking parlor or holding cows with the headgate closed before the feed is offered, are advised. However, the latter could stimulate the saliva production due to neuronal impulses if the feeding time is close to and, therefore, produce changes in the measured salivary analytes' concentration/activity; an effect to be evaluated in the future.

This study has some limitations. The results obtained in this report must be taken carefully since they are related to the cow population sampled and obtained under healthy status. Therefore, additional studies in other cow populations and diseases or stress situations must be performed to check whether these changes due to the feed presence produce a similar effect compared to the clean sample, and whether these variabilities could mask changes in salivary analytes due to these situations. Also, in our report the incubation in vitro lasted 5 min in order to take into consideration that many times feed can be around the buccal vestibule or under the tongue and persist mixed with saliva during approximately this time. However, the influence of other times of incubations has not been evaluated. Additionally, this results’ interpretation should not be extrapolated to another animal species since some analytes (i.e., gGT, or phosphorus) changed differently compared to horses when a similar experimental procedure was performed [13].

## Conclusions

In the conditions of this study, the cows' saliva contamination by a standard feed based on a total mixed ration, wheat hay, and grass can produce changes in the results of salivary analytes related to welfare and health status. Although saliva from diseased or stressed cows was not evaluated in this study, these changes could potentially have an influence on the interpretation of the analytes when applied as biomarkers. Therefore, ideally, it would be advised to use saliva samples in cows where mouths are as clean as possible to have consistent results. This could be obtained by washing their mouth or collecting the saliva sample before the feed is offered. In the case of being not possible, researchers should be aware that feed can affect selected salivary analytes measurements, and comparisons of results from different studies should take this fact into account.

## Materials and methods

### Cow population

Five Holstein-Friesians cows (age range = 2–8 years; body condition score range = 2.5–3) from a southeast Spain commercial dairy herd (38º2´ N, 1º15´W) were enrolled in this study. They were examined by an experienced veterinarian (PJV-M) and were considered being clinically healthy, with no sign of painful disorders (i.e., lameness, acute mastitis, or metritis) or discomfort after a physical examination, and with normal heart and respiratory rates (68.2 ± 3.37 and 32.4 ± 4.42, respectively). The dairy cows were in dry period (from 45 to 15 days to expected parturition time). They were housed in small groups of 20–25 cows in sand-bedded free stalls (1.2 stalls/cow). Their feeding ﻿was based on a far-off total mixed ration ﻿from − 50 d relative to calving to pre-calving time offered ad libitum at 08:30 am. ﻿ This mixed ration (supplementary material, B) was based on (% of DM) oat silage (36.65%), oat hay (31.41%), barley -bagasse- (20.94%), rapeseed by-product (rapeseed cake) (7.33%), corn -ground- (3.14%), and a mineral premix. Its nutrient composition (% of DM, DM = 52.0%) was of NE_L_ 1.21 Mcal/kg, ADF 33.90%, NDF 44.84%, crude protein 13.87%, soluble protein 4.74%, RDP 8.72%, RUP 5.15%. The farm is free of brucellosis, tuberculosis, Bovine Leukosis Virus, and pleuropneumonia. The cows were vaccinated for Bovine Viral Diarrhea, Infectious Bovine Rhinotracheitis, Bovine parainfluenza-3, and Bovine respiratory syncytial virus.

### Salivary sampling

The saliva samplings were performed from 09:30 am to 12:25 pm along five different days for each cow along December 2020 and by the same researcher. The average temperature and humidity were ﻿18.5 ± 2.33 °C and 65.4 ± 15.21%, respectively. The cows were driven to each free-stall headgate and held immobilized once closed. No feed was presented in the ground. This driving procedure did not suppose an extra stressful situation since they are used to having these handling procedures for their routine clinical examinations. Five minutes before the saliva was obtained, cows’ mouths were washed as previously described in horses [13] to obtain a clean sample with no feed interference. Then, a 5 × 2 × 2 cm polypropylene sponge (Esponja Marina, La Griega E. Koronis, Madrid, Spain) clipped to an independent flexible thin metal rod was introduced into the cow mouth vestibule and under de tongue until thoroughly moist since the parotid and sublingual salivary glands are the most developed [24]. Cows were previously accustomed to washing mouth and saliva collection by an earlier performance one day before the first saliva sampling.

Once the saliva was obtained, sponges were placed into a commercially available saliva device (Salivette, Sarstedt, Aktiengesellschaft & Co, Nümbrecht, Germany). They were kept on ice until arrival at the laboratory (less than one hour), where the tubes were processed as previously described [10]. Saliva specimens were stored at ﻿ − 80 ◦C until analysis (less than five months).

### In vitro* experiment*

**Fig. 3:**
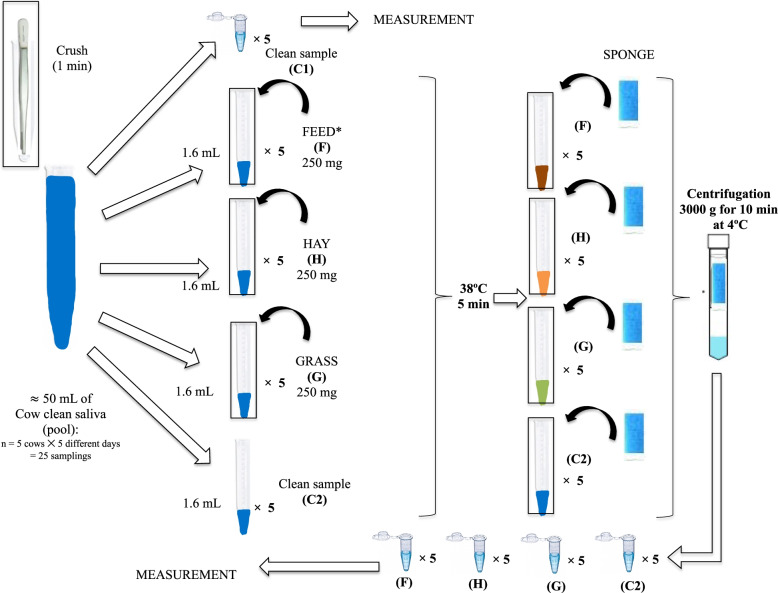
﻿In vitro experimental workflow performed for the food effect evaluation in a profile of salivary analytes measurement in pool saliva from five cows during five different days. *Standard feed based on a total mixed ration (broccoli -remains-, oat silage, corn -ground-, barley -bagasse-, alfalfa silage, wet corn pulp, rapeseed, dehydrated alfalfa, dry beet pulp, non-protein hydrogen source, barley straw, and a mineral pre-mix)

The clean saliva specimens previously obtained from the five cows were thawed and ﻿mixed in a pool (50 mL in total, approximately). Then, the clean saliva pool was divided into five specimens, and each one was additionally divided into ﻿ × 5 replicates (Fig. 3). Specimen C1 was defined as the clean saliva control and measured without any treatment. The additional four specimens were submitted to the followings treatments: 1.6 mL of clean saliva were placed into different standard polystyrene tubes with round bottoms (10 mL, ﻿16 × 95 mm) and mixed each one with 250 mg of a standard lactating cow feed (specimen F) based on broccoli -remains-, oat silage, corn -ground-, barley -bagasse-, alfalfa silage, wet corn pulp, rapeseed by-product (rapeseed cake), dehydrated alfalfa, dry beet pulp, non-protein hydrogen source, barley straw, and a mineral pre-mix (supplementary material, A), wheat hay (specimen H), and grass (specimen G), or without any contamination with feed (specimen C2 or clean saliva control 2). The feed mixed with saliva in the F, H, and G specimens was ﻿crushed with forceps for 1 min to simulate feed chewing. Then, these four specimens were ﻿incubated for 5 min at 38 °C (AccuTherm, Labnet International Inc., Madrid, Spain) since the normal adult cow rectal temperature range is 38.5–39.5 °C [25]. Finally, ﻿a polypropylene sponge was introduced in each polystyrene tube, and it was crushed for 1 min for the saliva to soak up the sponge and centrifuged as described above. The recovered saliva was then used for measuring each replicate. Each replicate from each specimen was obtained at the same time. Additionally, an identical experimental procedure was performed, replacing the saliva with ﻿deionized water for the specimens F (F_w_), H (H_w_), G (G_w_), and C2 (C2_w_).

### Analytical methods

The sialochemistry profile was integrated by:

- Stress biomarkers. sCor, sAA, BChE, TEA, and Lip.
- Immunity biomarkers. ADA.
- Oxidative status biomarkers. TEAC, FRAS, CUPRAC, uric acid, and AOPP.
- Biomarkers of general metabolism and liver, muscle, and renal damage (enzymes, proteins, and minerals). AST, ALP, gGT, LDH, CK, creatinine, urea, triglycerides, glucose, lactate, total protein, phosphorus, and total calcium.

All the analytes involved in this salivary profile were analyzed on an automated chemistry analyzer (Olympus Diagnostica GmbH AU 400, Beckman Coulter, Ennis, Ireland) using commercial kits from Beckman (Beckman Coulter Inc., Fullerton, CA, USA), except for BChE and TEA, which were measured according to previously reported assays [26, 27], ADA, which was measured with a Diazyme kit (ADA-D assay kit, Diazyme Laboratories, Poway, CA, USA); TEAC, FRAS, CUPRAC, and AOPP, which were assessed in saliva according to Rubio et al., [28], and total proteins, which it was measured by a ﻿commercial colorimetric kit to measure urine and cerebrospinal fluid (CSF) proteins (protein in urine and CSF, Spinreact, Spain). sCor was ﻿analyzed using a chemiluminescent immunoassay system (Immulite 1000, Siemens Healthcare Diagnostic, Deerfield, IL, USA). All these assays were adapted to saliva and previously validated in cow saliva [9, 10].

### Statistical analysis

To evaluate if there were differences in the values obtained between the specimens C1 (clean control saliva), C2, F, H, and G, a one-way repeated measures analysis of variance (ANOVA) and an uncorrected Fisher’s least significant difference (LSD) test for multiple comparisons was performed to determine what specimens showed statistically different values to C1 specimen. Additionally, another Fisher’s LSD test was calculated to analyze if there would be differences between the saliva specimens with their homologous with water (C2_w_, F_w_, H_w_, and G_w_). Data were checked for normality using the Shapiro–Wilk normality test previous to the analysis. The biomarkers showing non-normal distribution (BChE, ADA, uric acid, AST, creatinine, lactate, and total protein) were then base-e log-transformed [29] to restore normality. A *P*-value < 0.05 was considered as being statistically significant. The statistical analyses were calculated using Graph Pad Software Inc. (GraphPad Prism, version 9.1.0 for macOS; Graph Pad Software Inc., San Diego, CA, USA).

The CV (%) of the means from specimens C2, F, H, and G compared to C1 was calculated as the following formula:

$$\pm \frac{\mathrm{SD}\left(\overline{x }\mathrm{C}1, \overline{x }\mathrm{x}\right)\times 100}{\overline{x }\left(\overline{x }\mathrm{C}1, \overline{x }\mathrm{x}\right)}$$

where $\overline{x }$ C1 was the arithmetic mean from the × 5 replicates measured from specimen C1, and ${\overline{x} }_{\mathrm{X}}$the arithmetic mean from the × 5 replicates measured from specimens C2, F, H, or G. Positive ( +) or negative (–) CV value indicates that the average value obtained from specimens C2, F, H, or G were higher or lower from specimen C1, respectively.

CV higher than 15% between the mean values from each saliva treatment and the mean values obtained from specimen C1 (clean saliva) were considered high enough to show imprecise values [30]. Therefore, they were considered as specimens in which interference has occurred.

## Supplementary Information

**Additional file 1.**

## Data Availability

The datasets generated and/or analyzed during the current study are not public due to legal reasons but available from the corresponding authors on reasonable request.

## Abbreviations

sAA: Salivary alpha-amylase
sCor: Salivary cortisol
BChE: Butyrylcholinesterase
TEA: Total esterase
Lip: Lipase
ADA: Adenosine deaminase
TEAC: Trolox equivalent antioxidant capacity
FRAS: The ferric reducing ability of saliva
CUPRAC: The cupric reducing antioxidant capacity
AOPP: Advanced oxidation protein products
AST: Aspartate aminotransferase
ALP: Alanine aminotransferase
gGT: γ-Glutamyl transferase
LDH: Lactate dehydrogenase
CK: Creatine kinase
CV: Coefficient of variation
ANOVA: Analysis of variance
LSD: Least significant difference
