# Supplementary material for: Changes in salivary analytes in cows due to the in vitro presence of feed
Source: BMC Vet Res. 2022 Jul 14;18:275. doi: 10.1186/s12917-022-03371-9 (PMC9281046; doi:10.1186/s12917-022-03371-9)
Supplement: Supplementary file 1 — Additional file 1. [file 12917_2022_3371_MOESM1_ESM.docx]

|  | |  | |  | |
| --- | --- | --- | --- | --- | --- |
| Ingredient, % of DM |  |  | |  | |
| Broccoli (remains) |  |  | | 29.11 | |
| Oat silage | |  | | 20.38 | |
| Corn, ground | |  | | 14.56 | |
| Barley, bagasse |  |  | | 10.19 | |
| Alfalfa silage | |  | | 7.28 | |
| Wet corn pulp | |  | | 7.28 | |
| Rapeseed by-product (rapeseed cake)) | | cake) | | 2.91 | |
| Dehydrated alfalfa | |  | | 2.91 | |
| Dry beet pulp | |  | | 2.18 | |
| Non-protein hydrogen source | |  | | 1.75 | |
| Barley straw | |  | | 0.73 | |
| Mineral premix^a^ | |  | | 0.73 | |
|  |  |  | |  | |
| Nutrient composition, % of DM | | |  | |  |
| DM, % | |  | | 41.80 | |
| NE_L_, Mcal/kg | |  | | 1.51 | |
| ADF, % | |  | | 20.14 | |
| NDF, % | |  | | 32.06 | |
| Crude proteins, % | |  | | 14.39 | |
| Soluble protein, % | |  | | 5.21 | |
| RDP, % | |  | | 9.08 | |
| RUP, % | |  | | 5.31 | |

**Supplementary material.**

**A)** Ingredient and nutrient compositions of diet used for the experimental procedure. The information shows the ﻿base-ration offered to lactating cows.

^a^The vitamin-mineral premix composition was as follows: 292.34 g Ca, 107.35 g P, 63.69 g Mg, 66.16 g S, 399.27 g K, 91.22 g Na, 108.38 g Cl, 7.87 g Fe, 1.18 g Zn, 1.50 g Mn, 319.85 mg Cu, 14.93 mg I, 10.02 mg Co, 12.42 mg Se, 100 000 IU vitamin A, 4 000 IU vitamin D, and 100 mg vitamin E.

**B)** Ingredient and nutrient compositions of diet offered to the dry period cows used in the experimental procedure.

|  | |  |  |
| --- | --- | --- | --- |
| Ingredient, % of DM |  |  |  |
| Oat silage | |  | 36.65 |
| Oat hay | |  | 31.41 |
| Barley, bagasse |  |  | 20.94 |
| Rapeseed by-product (rapeseed cake) | |  | 7.33 |
| Corn, ground | |  | 3.14 |
| Mineral premix^a^ | |  | 0.52 |
|  |  |  |  |
| Nutrient composition, % of DM | | |  |
| DM, % | |  | 52.0 |
| NE_L_, Mcal/kg | |  | 1.21 |
| ADF, % | |  | 33.90 |
| NDF, % | |  | 44.84 |
| Crude proteins, % | |  | 13.87 |
| Soluble protein, % | |  | 4.74 |
| RDP, % | |  | 8.72 |
| RUP, % | |  | 5.15 |

^a^The vitamin-mineral premix composition was as follows: 58.71 g Ca, 44.19 g P, 32.39 g Mg, 26.17 g S, 167.72 g K, 28.33 g Na, 51.13 g Cl, 3.26 g Fe, 367.36 mg Zn, 861.53 mg Mn, 96.20 mg Cu, 7.67 mg I, 7.19 mg Co, 7.54 mg Se, 100 000 IU vitamin A, 4 000 IU vitamin D, and 100 mg vitamin E.
